# Return to driving following rotator cuff repair: 23%, 70% and 99% at 1, 2 and 6 months

**DOI:** 10.1002/jeo2.70201

**Published:** 2025-04-16

**Authors:** Maxime Antoni, Mohamed Zaibi, ReSurg, Floris Van Rooij, Bethany Grew, Florent Baldairon

**Affiliations:** ^1^ Clinique de l'Orangerie Strasbourg France; ^2^ ReSurg SA Nyon Switzerland; ^3^ Clinique du Sport Bordeaux‐Merignac France

**Keywords:** automatic car, driving, manual car, rotator cuff repair, rotator cuff tear

## Abstract

**Purpose:**

To report the rate and time of return to driving during the first 12 months following rotator cuff repair (RCR), and to determine the influence of preoperative factors on return to driving.

**Methods:**

The authors retrospectively collected the records of patients that underwent RCR for superior (isolated supraspinatus), anterosuperior (supraspinatus + subscapularis), posterosuperior (supraspinatus + infraspinatus), and 3‐tendon tears between 2021 and 2022. Patients were excluded if they had bilateral RCR, previous ipsilateral shoulder surgery, or any discomfort at the contralateral shoulder, and a total of 151 patients (151 shoulders) met the inclusion criteria. Of the initial cohort of 151 patients, six did not drive, eight required reoperation, and nine were lost to follow‐up. This left a final cohort of 128 patients aged 59.1 ± 7.6 years (range, 35–79), of which most were males (61%), and drove manual cars (70%). Most patients had a superior tear (53%) and had their right side operated (69%). The authors noted patient demographics, surgical data, and pre‐ and postoperative driving details, as well as the Constant score, American Shoulder and Elbow Surgeons (ASES) score, and Subjective Shoulder Value (SSV) at 6 and 12 months.

**Results:**

Following rotator cuff repair 23% of patients returned to driving at 1 month, 70% at 2 months and 99% at 6 months. The time to return to driving less than 5 km of 7.3 ± 4.8 weeks (range, 0.5–26). Univariable analysis revealed that earlier return to driving was associated with male sex (*β*, −2.609; *p* = 0.002), automatic cars (*β* −2.050, *p* = 0.027) and distance driven per week (*β*, −0.004, *p* = 0.043).

**Conclusions:**

Rotator cuff repair enables 23% of patients to return to driving at 1 month, 70% at 2 months, and 99% at 6 months. Male sex, patients with automatic cars, and distance driven per week preoperatively were indicators for earlier return to driving.

**Level of Evidence:**

Level IV, retrospective case series.

**Abbreviations**
ASES: American Shoulder and Elbow Surgeons
RCR: rotator cuff repair
SSV: subjective shoulder value

## INTRODUCTION

Many surgeons recommend driving restrictions for 4–6 weeks following rotator cuff repair (RCR) as patients require the use of a sling [1, 12, 13]. However, as most patients require driving to perform activities of daily living, a common question is “when can I drive following RCR?” A recent editorial highlighted the lack of evidence in this field [10], making it difficult for surgeons to advise patients appropriately, as it involves patient, surgeon, social and legal considerations [2, 11].

In 2016, a meta‐analysis evaluated return to driving following orthopaedic procedures, but only included one study on RCR, that reported an average time to return to driving of 2 months [5]. A recent study by Badger et al. [1] evaluated driving capabilities following RCR in a cohort of 32 patients, and found, using kinematic analysis, non‐inferiority of all post‐operative driving manoeuvres compared to baseline, including left and right turns, highway merges and yielding onto oncoming traffic. Finally, Badger et al. concluded that there was no significantly negative impact on driving capability as early as 2 weeks after RCR. Recently, a protocol for a scoping review was registered [15], which may synthesise the literature. Due to limited evidence, it is unclear what the rate and time to return to driving is in a larger cohort, which could leave doubts for surgeons when questioned by patients on when they can return to driving.

Therefore, the purpose of the present study was to report the rate and time of return to driving during the first 12 months following RCR, and to determine the influence of preoperative factors on return to driving. The findings of this study could assist surgeons in making recommendations to patients and provide information on when patients return to driving following RCR. The hypothesis was that the majority of the patients would return to driving at 12 months follow‐up.

## MATERIALS AND METHODS

**Figure 1:**
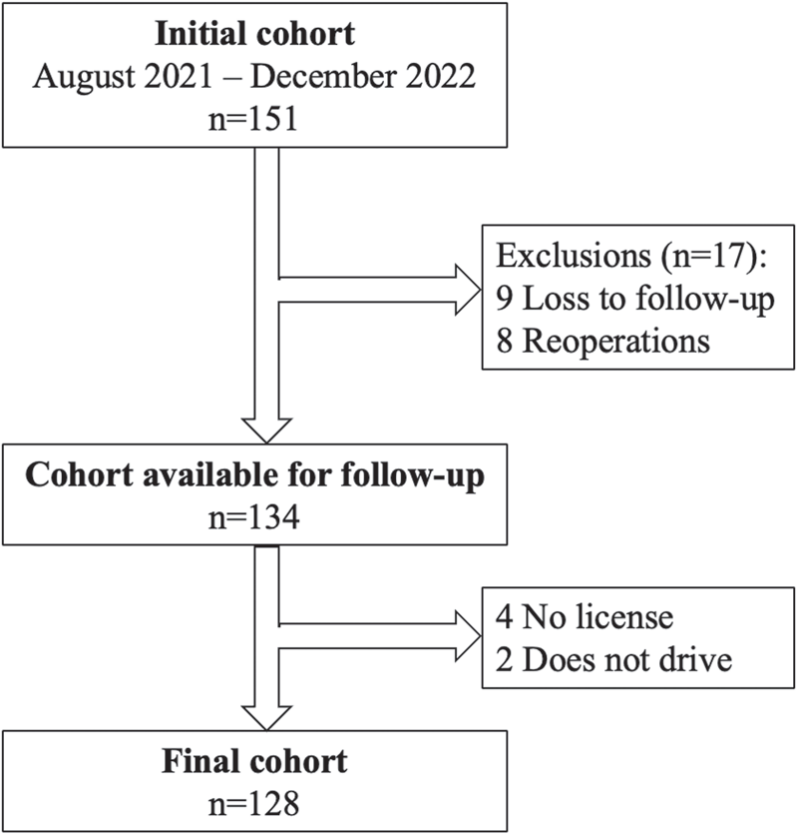
Flowchart.

The authors retrospectively reviewed prospectively collected data of patients that underwent RCR for superior tears (isolated SSP), anterosuperior tears (SSP + SSC), posterosuperior tears (SSP + ISP), and 3‐tendon tears from August 2021 to December 2022 at two centres. All patients provided informed consent for their participation in this study, which had been approved by the institutional review board in advance (#2024‐03‐Dr ANTONI‐01). Patients were excluded if they were over 80 years, did not have a driver's license, had been in a car accident in the year prior, had bilateral RCR, previous ipsilateral or revision shoulder surgery, or any discomfort at the contralateral shoulder. A total of 145 patients (145 shoulders) met the criteria for inclusion. From the initial cohort, eight required reoperation (6%), and nine were lost to follow‐up (6%), which left a total of 128 patients (Figure 1; Table 1).

**Table 1:** Demographics and perioperative data.

	Final cohort (*n* = 128)		
	Mean ± SD		
	*n* %		Range
Age	59.1 ± 7.6		(35–79)
Female sex	50 39%		
Drives			
Residence			
Urban	61 48%		
Rural	67 52%		
Work status			
Working	82 64%		
Retired	45 35%		
Unable to work	1 1%		
Operating centre			
Public hospital	48 38%		
Private clinic	80 63%		
Right side operated	88 69%		
Tear pattern			
Anterosuperior (SSP + SSC)	18 14%		
Posterosuperior (SSP + ISP)	25 20%		
Superior (isolated SSP)	68 53%		
Three tendon	17 13%		

Abbreviations: ISP, infraspinatus; SD, standard deviation: SSC, subscapularis; SSP, supraspinatus.

### Preoperative assessment

The authors retrieved patient demographics, location of residence (urban/rural), work status (working/retired/unable to work), operating centre (private clinic/public hospital), side operated, and tear pattern. In addition, the authors noted patient driving details, including whether the patient drives, years of having license, whether they were the only driver in the household, need to drive children, type of car (automatic/manual), and distance driven per week (km).

### Surgical technique

All patients underwent arthroscopic RCR in the beach chair position under general anaesthesia, with an interscalene block. Biceps tenotomy was performed in all cases, followed by tenodesis if required. First, a debridement of the tendon was performed, followed by debridement of the footprint using a burr. The tendons were reattached using a single or double row method, depending on the size of the tear and its retraction. Finally, acromioplasty was performed with a burr to improve exposure and to reduce impingement with the cuff.

### Postoperative rehabilitation

All patients required shoulder immobilisation with a sling for 4 weeks postoperatively and started self‐rehabilitation exercises Day 1 after surgery following a standard protocol (consisting of pendulum exercises, and gentle assisted flexion), followed by a rehabilitation programme supervised by a physiotherapist, where patients focus on further mobilisation and external rotation can be performed, however this is up to the discretion of the physiotherapist. Patients were allowed to return to daily activities after 1 month, and lifting heavy objects (approximately 2 kg, the weight of big bottle of water) after 3 months. Patients were allowed to return to driving, regardless of time since index surgery, if (i) they were not (or no longer) using a sling AND if (ii) they had a low level of pain with sufficient ROM. All patients were prescribed opioids for 3 days following surgery, and use of further painkillers were left to the discretion of the patient.

### Postoperative evaluation

The surgeon performed the clinical assessment at 6 and 12 months using the Constant score [3], American Shoulder and Elbow Surgeons (ASES) score [16] and the Subjective Shoulder Value (SSV) [6, 8]. At 12 months, the primary outcome was to assess whether patients returned to driving, as well as at what timepoint they returned to driving less than 5 km and more than 30 km (these distances were chosen arbitrarily by the surgeon based on the distance to reach shops, or to travel further), and at what timepoint they returned to driving without discomfort. The secondary outcome was to determine to influence of independent variables on time to return to driving.

### Statistical analysis

Descriptive statistics were used to summarise demographic data, surgical data, driving details and clinical scores. Uni‐ and multivariable linear regression analyses were performed to determine the influence on time to return to driving of 12 independent preoperative variables (age, sex, location of residence, work status, operating centre, side operated, tear pattern, years of having license, only driver in household, type of car, need to drive children and distance driven per week). Multivariable logistic regression analyses were performed after selection of pertinent variables using directed acyclic graphs (DAG) (Supporting Information: Appendix 1). Statistical analysis was performed using R version 3.4.3 (R Foundation for Statistical Computing, Vienna, Austria). *p*‐Values < 0.05 were considered statistically significant.

## RESULTS

### Preoperative driving details

The 128 patients had a license for 37.4 ± 9.7 years (range 7–59) (Table 2), 35 were the only driver in their household (27%), 12 were required to drive children (9%), and 89 had a manual car (70%). The distance driven per week preoperatively was 221.7 ± 238.4 km (range 10–1000).

**Table 2:** Preoperative driving details of final cohort (*n* = 128).

	Mean ± SD				
	*n* %		Range	Median	IQR
Years of having license	37.4 ± 9.7		(7–59)	*38.5*	*(31–44)*
Only driver in household	35 27%				
Need to drive children	12 9%				
Type of car					
Automatic	39 30%				
Manual	89 70%				
Distance driven per week (km)	221.7 ± 238		(10–1000)	*150.0*	*(50–300)*

Abbreviations: IQR, interquartile range; SD, standard deviation.

### Postoperative driving details and clinical outcomes

At 12 months follow‐up, 127 patients had returned to driving (99%) (Table 3). The time to return to driving less than 5 km was at a mean of 1.7 ± 1.1 months (range, 0.1–6.0); 70% returned to driving at 2 months, and 99% at 6 months. Furthermore, the time to return to driving more than 30 km was at a mean of 2.5 ± 1.6 months (range, 0.1–9.2); 45% returned to driving at 2 months, and 98% at 6 months. The time until able to drive without discomfort was at a mean of 4.2 ± 2.8 months (range, 0.9–12.0), while nine patients still had discomfort while driving at 1 year (7%). Finally, none of the patients were involved in any driving accident.

**Table 3:** Postoperative driving details and clinical outcomes (*n* = 128).

	Mean ± SD				
	*n* %		Range	*Median*	*IQR*
Driving details					
Time to return to driving < 5 km	1.7 ± 1.1		0.1–6.0	*1.4*	*(1.2–2.1)*
1 month	30 23%				
2 months	90 70%				
6 months	127 99%				
12 months	127 99%				
Did not return to drive at 1 year	1 1%				
Time to return to driving >30 km	2.5 ± 1.6		0.1–9.2	*2.1*	*(1.4–3.0)*
1 month	17 13%				
2 months	58 45%				
6 months	125 98%				
12 months	127 99%				
Did not return to drive at 1 year	1 1%				
Time until able to drive without discomfort	4.2 ± 2.8		0.9–12.0	*3.0*	*(2.3–5.4)*
1 month	2 2%				
2 months	24 19%				
6 months	99 77%				
12 months	118 92%				
Still had discomfort at 1 year	9 7%				
Did not return to drive at 1 year	1 1%				
Clinical scores at 6 months					
Constant	62.8 ± 15.5		21–100	*65.0*	*(51.8–73.0)*
ASES	66.9 ± 16.4		17–100	*67.0*	*(53.0–78.5)*
SSV	70.5 ± 16.2		30–100	*70.0*	*(60.0–80.0)*
Clinical scores at 12 months					
Constant	73.8 ± 14.6		14–100	*76.5*	*(67.8–85.0)*
ASES	78.1 ± 17.1		13–100	*85.0*	*(67.8–90.0)*
SSV	81.1 ± 16.6		20–100	*85.0*	*(73.8–95.0)*

Abbreviations: ASES, American Shoulder and Elbow Surgeons score; IQR, interquartile range; SD, standard deviation; SSV, Subjective Shoulder Value.

At 12 months, the Constant score was 62.8 ± 15, the ASES was 66.9 ± 16.4, and the SSV was 70.5 ± 16.2.

Furthermore, there were no differences in outcomes for patients with anterosuperior, posterosuperior, superior, or 3‐tendon tears (Table 4).

**Table 4:** Postoperative driving details and clinical outcomes stratified by tear pattern.

	Anterosuperior	Posterosuperior	Superior	3‐Tendon	
	Mean ± SD	Mean ± SD	Mean ± SD	Mean ± SD	
	*n* %	*n* %	*n* %	*n* %	*p* value
Driving details					
Time to return to driving <5 km (weeks)	7.58 ± 6.32	7.16 ± 4.02	7.75 ± 5.04	5.65 ± 3.00	0.556
Time to return to driving >30 km (weeks)	10.08 ± 7.57	10.84 ± 5.82	11.23 ± 7.72	8.65 ± 6.02	0.559
Time until able to drive without discomfort	15.00 ± 11.35	21.08 ± 12.73	18.51 ± 12.97	15.41 ± 9.72	0.317
Clinical scores at 6 months					
Constant	66.61 ± 12.69	62.88 ± 13.34	61.35 ± 17.21	64.41 ± 14.26	0.572
ASES	69.56 ± 12.61	69.00 ± 15.51	65.07 ± 17.86	68.06 ± 15.09	0.647
SSV	70.56 ± 15.23	71.20 ± 16.28	70.07 ± 16.78	70.88 ± 16.22	0.968
Clinical scores at 12 months					
Constant	78.11 ± 12.41	73.52 ± 10.93	72.75 ± 16.99	74.18 ± 10.96	0.413
ASES	80.28 ± 17.91	80.52 ± 14.27	75.87 ± 18.66	81.00 ± 13.44	0.621
SSV	82.22 ± 17.92	83.60 ± 12.63	79.75 ± 18.40	80.59 ± 13.10	0.827

Abbreviations: ASES, American Shoulder and Elbow Surgeons score; SD, standard deviation; SSV, Subjective Shoulder Value.

### Regression analysis

Multivariable analysis revealed that men returned to driving earlier than women (*β*, −2.609; *p* = 0.002), patients with automatic cars returned to driving earlier than those with manual cars (*β*, −2.050, *p* = 0.027), and those that drive more per week (*β*, −0.004, *p* = 0.043). Other preoperative variables including age, residence, work status, operating centre, side operated, number of tendons torn, tear pattern, years of having license, only driver in household, and need to drive children were not found to have a significant influence on time to return to driving (Table 5).

**Table 5:** Regression analyses to identify factors associated with time to return to driving (*n* = 128).

	**Univariable**			**Multivariable**		
		95% C.I.			95% C.I.	*p* value
	*β*	(range)	*p* value	*β*	(range)	
Age	−0.010	(−0.12 to 0.10)	*0.862*	−0.010	(−0.12 to 0.10)	*0.862*
Male sex	−2.609	(−4.36 to −0.98)	***0***. ***002***	−2.609	(−4.36 to −0.98)	***0***. ***002***
Residence						
Rural	REF			REF		
Urban	1.095	(−0.60 to 2.79)	*0.204*	1.095	(−0.60 to 2.79)	*0.204*
Work status						
Working	REF			1.254	(−1.23 to 3.74)	*0.320*
Retired	−0.753	(−2.54 to 1.04)	*0.406*	REF		
Unable to work	1.414	(−8.27 to 11.09)	*0.773*	2.632	(−7.25 to 12.52)	*0.599*
Operating centre						
Private clinic	REF			0.280	(−1.5 to 2.1)	*0.759*
Public hospital	0.004	(−1.75 to 1.76)	*0.996*	REF		
Right side operated	0.191	(−1.65 to 2.03)	*0.837*	0.191	(−1.65 to 2.03)	*0.837*
Number of tendons torn						
1	REF			REF		
2	−0.417	(−2.28 to 1.45)	*0.659*	−0.417	(−2.28 to 1.45)	*0.659*
3	−2.107	(−4.70 to 0.49)	*0.110*	−2.107	(−4.70 to 0.49)	*0.110*
Tear pattern						
Superior	REF			−1.766	(−6.98 to 3.45)	*0.504*
Anterosuperior	−0.058	(−2.54 to 2.43)	*0.964*	REF		
Posterosuperior	−1.078	(−3.08 to 0.93)	*0.289*	−0.423	(−3.39 to 2.54)	*0.778*
Three tendon	−1.994	(−4.54 to 0.55)	*0.123*			
Years of having license	−0.040	(−0.13 to 0.05)	*0.369*	−0.099	(−0.25 to 0.05)	*0.196*
Only driver in household	−0.930	(−2.83 to 0.97)	*0.335*	−0.930	(−2.83 to 0.97)	*0.335*
Type of car						
Manual	REF			REF		
Automatic	−2.050	(−3.86 to −0.24)	***0***. ***027***	−2.050	(−3.86 to −0.24)	***0*.*027***
Need to drive children	−0.549	(−3.46 to 2.36)	*0.710*	−0.549	(−3.46 to 2.36)	*0.710*
Distance driven per week (km)	−0.003	(−0.01 to 0.00)	*0.058*	−0.004	(−0.01 to 0.00)	***0***. ***043***

*Note*: Bold values indicate statistically significant findings.

Abbreviation: C.I., confidence interval.

## DISCUSSION

The most important findings of the present study were that at a follow‐up of 12 months following RCR, 99% of patients had returned to driving, with an average time to return to driving less than 5 km of 7.3 ± 4.8 weeks. Furthermore, regression analysis revealed that earlier return to driving was associated with male sex, automatic car use, and with higher distance driven per week. The findings of this study could assist surgeons to make evidence‐based recommendations to patients on when patients could return to driving following RCR, and what factors might influence it.

There is limited evidence on rates of return to driving following RCR; in 2015, Gholson et al. [7] surveyed 54 patients on return to driving following RCR and found that only two patients did not return to driving at 4 months follow‐up. Furthermore, Gholson et al. [7] found that return to driving varied widely from the day of surgery to 4 months postoperatively, with the median being 2 months, which is slightly longer than the present study's median of 6 weeks to return to driving <5 km. Gholson et al. [7] also looked at other factors contributing to patients' perceptions of safety and manoeuvrability while driving following RCR; shoulder pain and weakness were associated with feeling unsafe while driving and difficulty manoeuvring, however this did not impact their driving capability. The present study did not investigate drivers' perceptions of safety and manoeuvrability, and hence further high‐quality studies are needed to confirm the results by Gholson et al. [7].

Although the present study found that some patients return to driving as early as a few days postoperatively, this raises questions about the safety of returning to driving in the early postoperative period following RCR. In 2015, Hasan et al. [9] investigated using a driving simulator in 21 healthy volunteers to compare the driving ability with vs without a sling on their dominant arm, and found that the sling group had a significantly higher amount of collisions compared to those without a sling (3.7 vs. 1.7, *p* < 0.01). The study by Hasan et al. used a simulator of an automatic transmission vehicle in healthy volunteers, which may suggest that use of a manual transmission vehicle in patients recently operated could have more dangerous consequences. Of note, the majority of patients in the present study drove manual vehicles (70%). Nonetheless, in 2022 Badger et al. [1] evaluated driving capability following RCR using subjective and objective assessments of driving performance, and found no significantly negative impact on driving capability as early as 2 weeks after RCR. Badger et al. [1] found that, from 2 weeks postoperatively, patients braked less aggressively, steered more smoothly, and drove more stably, suggesting that patients tend to drive more cautiously following RCR which may compensate for shoulder limitations [10].

A recent study published in 2023 by DeBernardis et al. [4] compared return to driving after anatomic versus reverse shoulder arthroplasty, and defined early return to driving as <2 weeks postoperatively. DeBernardis et al. found increased odds of early return to driving after anatomic shoulder arthroplasty in male patients (*p* = 0.002) and those instructed to drive following cessation of sling use (*p* = 0.024) or cessation of narcotics (*p* = 0.009), while following reverse shoulder arthroplasty, the odds increased with greater age (*p* = 0.019), higher BMI (*p* = 0.008), and those instructed to drive following cessation of sling use (*p* = 0.004). Unfortunately, DeBernardis et al. [4] did not include type of car (automatic or manual) in their regression analysis, making it difficult to compare to the present study; which revealed that male patients returned to driving earlier than females, and those with automatic cars returned earlier than those with manual cars. Finally, a recent meta‐analysis and clinical study found that following RCR, women had lower shoulder ROM and poorer outcomes than men, which could influence return to driving rates [14, 17].

The present study had a number of limitations. First, the authors did not collect any preoperative clinical scores, making it difficult to compare to the literature and evaluate the net change in clinical scores. Second, even though all patients were asked to remember to note when they returned to driving, it is possible that some did not recall the exact timepoint, which may change the time they returned to driving. Third, there was no blinded evaluation, which may have introduced bias in the results of this study. Fourth, this study did not investigate the outcomes of tenodesis or tenotomy on the return to driving, as several studies found no differences in clinical outcomes. Finally, the authors did not assess driving metrics or ability to manoeuvre a car, could therefore not objectively quantify reaction times and car control.

## CONCLUSION

At a follow‐up of 12 months following RCR, 99% of patients had returned to driving, with an average time to return to driving less than 5 km of 7.3 ± 4.8 weeks. Regression analysis revealed that earlier return to driving was associated with male sex, automatic car use, distance driven per week. The findings of this study could assist surgeons in making recommendations to patients and to provide realistic expectations on when patients could return to driving following RCR, and what factors might influence it.

## AUTHOR CONTRIBUTIONS

**Maxime Antoni**: Conceptualization; methodology; funding acquisition; supervision. **Mohamed Zaibi**: Conceptualization; formal analysis and investigation. **Floris Van Rooij**: Formal analysis and investigation; writing–review and editing; supervision. **Bethany Grew**: Formal analysis and investigation; writing–original draft preparation. **Florent Baldairon**: Conceptualization.

## CONFLICT OF INTEREST STATEMENT

Maxime Antoni reports consulting fees from FX Shoulder Solutions and Pixee Medical. Floris Van Rooij, Bethany Grew, Mohamed Zaibi, and Florent Baldairon report no conflicts of interest.

## ETHICS STATEMENT

All patients provided informed consent for the use of their data for research and publications and the institutional review board approved this study in advance (#2024‐03‐Dr ANTONI‐01).

## Supporting information

Supporting information.

## Data Availability

All data is available upon reasonable request.
