# Supplementary figures and images for: Return to driving following rotator cuff repair: 23%, 70% and 99% at 1, 2 and 6 months
Source: J Exp Orthop. 2025 Apr 16;12(2):e70201. doi: 10.1002/jeo2.70201 (PMC12002080; doi:10.1002/jeo2.70201)

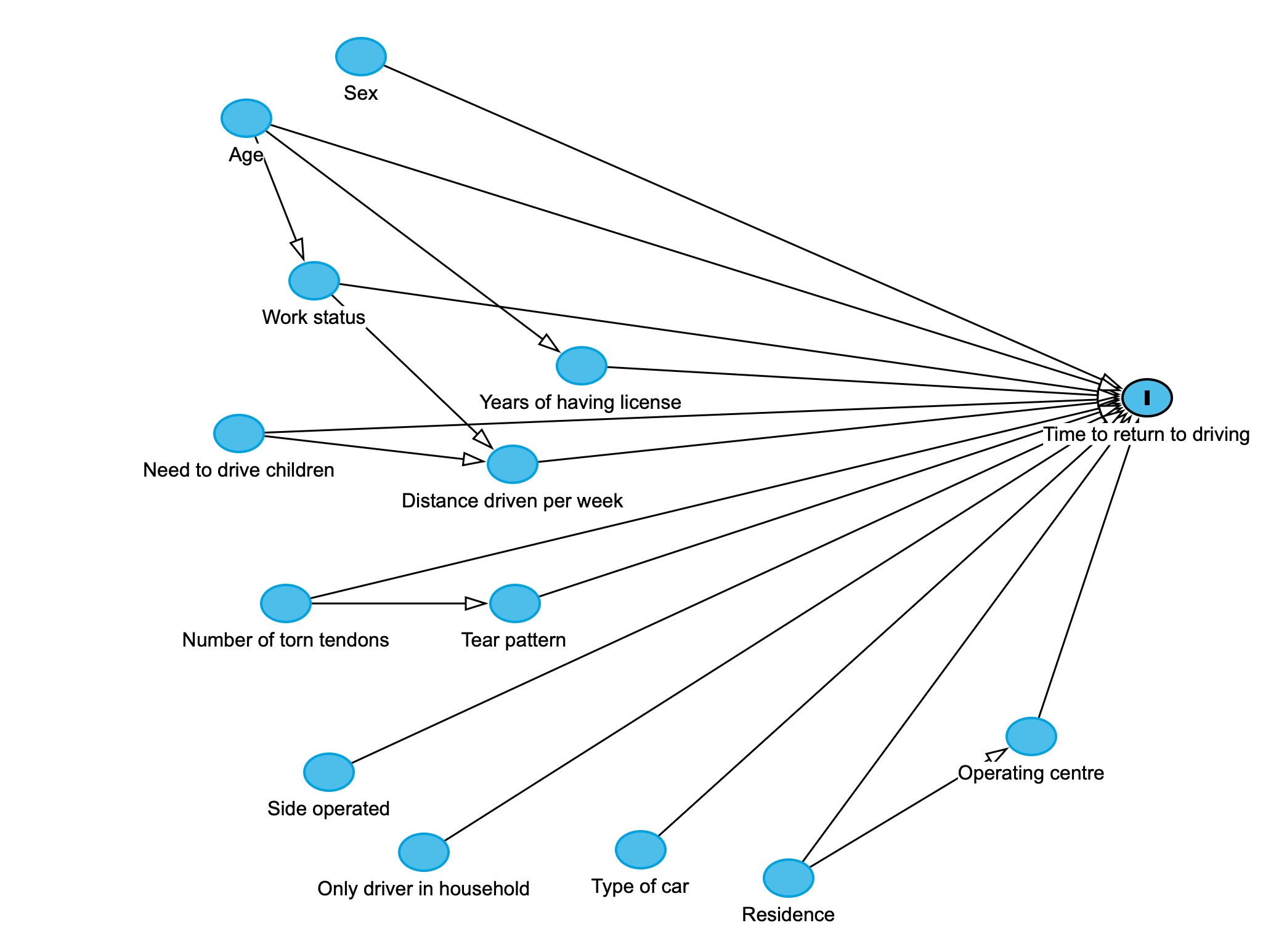

Supplement: Supplementary file 1 — Supporting information. [file JEO2-12-e70201-s001.jpeg]
